# Gnotobiotic Human Colon *Ex Vivo*

**DOI:** 10.14740/gr675w

**Published:** 2015-10-21

**Authors:** Frank D. McDermott, David M. A. Folan, Des C. Winter, Michael A. Folan, Alan W. Baird

**Affiliations:** a UCD School of Veterinary Medicine & Conway Institute of Biomolecular & Biomedical Science, University College Dublin, Belfield, Dublin 4, Ireland; b UCD School of Medicine and Medical Science, University College Dublin, Belfield, Dublin 4, Ireland; c Department of Surgery, St Vincent’s University Hospital, Elm Park, Dublin 4, Ireland; d These authors contributed equally to the study.

**Keywords:** Antiseptic, Gut bacteria, Ion transport, Permeability, Colonic epithelium

## Abstract

**Background:**

A novel emulsion with efficacy as an agent for eliminating biofilms was selected. The aim of this study was to examine efficacy and effect of a formulation of ML:8 against commensal bacteria harvested from *ex vivo* human colonic tissues.

**Methods:**

Mucosal sheets, obtained at the time of surgery, were exposed for 2 minutes to one of four solutions: Krebs-Hensleit (KH) solution, saline (NaCl; 0.9%), povidone iodine (1%), or ML:8 (2%); n = 4. Lumenal surfaces were swabbed for culture under aerobic or anaerobic conditions. Following treatment, each sheet was mounted in Ussing chambers and voltage clamped. Tissues were challenged with carbachol. Permeability coefficient (Papp) was determined using mannitol fluxes. At the end of each experiment, tissues were examined histologically.

**Results:**

Similar colony forming units grew in aerobic and anaerobic conditions in both control and NaCl treated tissues. Iodine reduced and ML:8 virtually abolished viable bacteria. Basal electrophysiological parameters were not different between treatments. Transepithelial electrical resistance values did not differ between groups. All tissues responded to carbachol, although this was attenuated in iodine treated tissue. Papp values were slightly elevated in all treated tissues but this did not reach significance. Histopathological assessment revealed no overt damage to tissues.

**Conclusion:**

Brief exposure to ML:8 reduced culturable bacterial burden from human intestinal tissues harvested at the time of surgical resection. Such gnotobiotic tissues retain structural and functional integrity. This is a novel approach to reduce bacterial burden.

## Introduction

*In vitro* studies of isolated intestine offer a reductionist approach to study interactions between a range of cell types in a spatially organized tissue. For example, isolated sheets of human colonic mucosa have been widely used to examine various aspects of physiology, pharmacology and pathology [1]. Mucosal sheets, stripped of underlying smooth muscle, conserve complex tissue architecture. Thus, free from neuronal, humoral or other extrinsic factors, a range of functions may be conveniently studied under voltage clamped conditions in Ussing chambers in the absence of transepithelial diffusion forces (chemical, hydraulic, osmotic or electrical gradients) [2]. The primary advantage of this *ex vivo* approach is to focus on specific functions or components in human tissues which is particularly significant since responses are often species-specific.

Mucosal interactions with bacteria have been examined using isolated mucosal sheets or cultured monolayers of epithelia [3]. Mucosal sheets from humans or experimental animals are non-sterile. Normally, the mammalian intestine is populated by enormous numbers and ranges of microbes which contribute to health and/or disease [4-6]. Major advances in microbiome analysis continue to inform our understanding of commensalism, symbiosis, pathogenesis and therapy [7-9]. The balance is dynamic and manipulation of the human intestinal microbiome by diet, antibiotics or disease is a common occurrence. Experimental investigation of the interactions of microorganisms with native, isolated epithelium is complicated by the existing burden, the microbiome. To overcome this, sterile sheets of epithelial cells in culture or tissues isolated from gnotobiotic animals have been used with some success [3, 10-12]. However, particularly since commensal bacteria contribute to “normal” homeostasis [13], specimens from “germ-free” animals are compromised and those obtained as biopsy or resection from humans are already host to a wide variety of microorganisms.

In order to achieve a novel experimental preparation - the gnotobiotic human intestine, we have attempted to reduce or remove the bioburden in sheets of mucosa which are obtained at the time of surgical resection. Specifically, we have employed sheets of human colon *ex vivo* to compare and contrast consequences of pre-exposure to three interventions. Two of these, saline and dilute iodine, are widely used as surgical irrigants as well as for wound irrigation and intra-cavity lavage [14]. These are all antibiotic-free although ampicillin, polymyxin and/or neomycin are often used clinically [15]. The third is an emulsion which is based upon a unique combination of free fatty acids, triglycerides and phospholipids.

The specific aims of this study were to examine the influence of brief exposure (2 min) of isolated sheets of human colonic mucosa to the three test solutions or to physiological solution as a control. Following exposure, each mucosal surface was swabbed for subsequent microbiological analysis. We selected a system for aerobic and also anaerobic cultures which has been validated as equivalent to standard plating methodology [16]. The mucosal sheets were then mounted in Ussing chambers and voltage clamped. Parameters which were measured, and compared between treatment groups, included transmucosal potential difference (PD), short circuit current (SCC), electrical resistance (R) and permeability to mannitol (Papp). Ion transport responses to carbachol were also compared between treatment groups. Finally, tissues were evaluated histologically.

## Materials and Methods

### Colon samples

Institutional Review Board approval (St Vincent’s University Hospital, Dublin, Ireland) including written informed patient consent was obtained. There were no cases where a parent or guardian was required to provide consent. Macroscopically normal specimens (left-sided, descending colon) were acquired at surgical resection for colonic carcinoma and immediately transferred to the laboratory in pre-oxygenated Krebs-Hensleit (KH) solution (in mM: NaCl 118, KCl 4.7, CaCl 2.5, MgSO_4_ 1.1, KH_2_PO_4_ 1.2, NaHCO_3_ 25, and D-glucose 11.1; pH 7.4). Three separate samples were obtained from each tissue and exposed to one of the three treatments for a 2-min incubation period before washing in sterile KH solution.

### Ussing chamber experiments

The sero-muscular layer was dissected off and the mucosa mounted into Ussing chambers (0.63 cm^2^ aperture; World Precision Instruments, Stevenage, UK), bathed in equal volumes (5 mL) of KH solution bilaterally. The buffer was continually perfused with 95% oxygen 5% carbon dioxide gas lift, and a thermostat-regulated circulating pump was used to maintain the temperature at 37 °C. Electrophysiological parameters were calculated and recorded as described previously [17]. Briefly, transepithelial PD (mV) was measured in the open circuit configuration after which the tissue was voltage clamped to zero PD by application of the required SCC (μA/cm^2^) by means of an automatic voltage clamp (EVC-4000 amplifier, AD Instruments, UK). SCC and PD were monitored by switching to open circuit conditions for 3 s every 30 s using a Pro-4 timer (AD Instruments, UK). Analogue data were digitized with a Powerlab^®^ data acquisition unit and analyzed with Chart^®^ software package (AD Instruments, UK). Following an equilibration period of 45 min, baseline PD and SCC were measured and R (Ω.cm^2^) was calculated according to Ohm’s law. The tissues capacity to generate an inward SCC response to basolateral addition of the muscarinic agonist, carbachol (0.1 - 10 μM), was used as a measure of epithelial function at the end of each experiment [18].

Permeability of voltage clamped mucosal sheets was determined by mannitol flux. Briefly, ^14^C-mannitol (0.2 μCi/mL) was added to the apical chamber and flux was monitored by sampling the basolateral chamber (100 μL) every 10 min for 2 h, and apically (200 μL) at time zero, replenishing with fresh KH buffer at each sampling point. Samples containing ^14^C-mannitol were mixed with scintillation fluid and read in a scintillation analyzer (Packard C Tricarb 2900 TR). The apparent permeability coefficients (Papp) were calculated according to the equation Papp (cm/s) = (dQ/dt)(1/AC_0_), where dQ/dt is the transport rate (mol/s); A is the surface area of the tissue section (cm^2^), and C_0_ is the initial concentration in the donor compartment (mol/mL) [19]. Tissues were fixed and stored in formalin for subsequent histopathological assessment.

### Bacterial sampling

Swabs were taken by lightly brushing the mucosal surface of individual samples of tissue after brief (2 min exposure) to either PBS, KH solution, iodine or ML:8. Aerobic and anaerobic bacteria were cultured separately (3M^TM^ Petrifilm^TM^ Count Plates 6400) incubated at 37 °C either in air or in anaerobic jars (Anaerogen, Oxoid, UK). Bacterial colonies on five separate, non-contiguous squares (each 1 cm^2^) were counted on each plate and an average was obtained. Counting was performed blindly by operators with no knowledge of the experimental groupings.

### Histology

Tissues removed from the Ussing chambers at the end of each experiment were fixed in formalin and semi-thin sections (5 μm) were cut on a microtome (Leitz 1512; GMI, USA), mounted on adhesive coated slides, and stained with hematoxylin and eosin (H&E). The slides were visualized using a light microscope (Labophot-2A; Nikon, Japan) and images were taken with a high-resolution camera (Micropublisher 3.3 RTV; QImaging, Canada) and Image-Pro^®^ Plus version 6.3 software (Media Cybernetics Inc., USA) [20].

### Formulation of ML:8 emulsion

ML:8 is a proprietary formulation consisting of 10% W/W compendial grade caprylic acid (Merck, Nottingham, UK), emulsified in Lipoid S75 lecithin (Lipoid AG, Steinhausen, Switzerland), and a water soluble block copolymer, Pluronic F68 (BASF , Cork, Ireland). The emulsion was formed in a high pressure Emulsiflex homogenizer operating at 1,000 bar and then dispersed at 2% w/w in sodium citrate buffer pH 5.0 and a physiological tonicity (280 mOsM). The ratio of free fatty acids to lipid was 8:1 and the final formulation was diluted 1 in 50 (0.2% w/w free fatty acids) in sterile water and autoclaved before use.

### Chemicals

All reagents were of ANALAR grade and purchased from Sigma-Aldrich (Dublin, Ireland). De-ionized water was used to make stock solutions of carbachol. Stock solutions were diluted to working concentrations in physiological solution (KH). Povidone iodine solution (Videne^®^, 2-pyrrolidinone, 1-ethenyl-, homopolymer with iodine) was obtained from Ecolab (Northwich, Cheshire, UK).

### Statistical analysis

Results are expressed as mean ± SEM where each n value represents a biological replicate, obtained from a different surgical procedure. Up to eight preparations were used to obtain matched, or paired test and control experiments from the same resection. Statistical analysis was carried out using GraphPad Prism (San Diego, CA, USA) using either the Mann-Whitney test or analysis of variance (ANOVA) to compare concentration-response curves. P values < 0.05 were considered statistically significant.

## Results

### Electrophysiology

Baseline electrophysiological parameters are shown in Table 1.

**Table 1:** Baseline Electrophysiological, Resistance and Papp Values for Human Colonic Tissue Mounted in Ussing Chambers (Mean Average ± SEM)

	SCC (μA/cm^2^)	PD (mV)	R (Ω.cm^2^)	Papp (cm/s)
Krebs-Hensleit solution (control) (n = 5)	-34.2 (± 6.0)	-5.8 (± 2.4)	118.5 (± 29.2)	0.82 ± 0.2 × 10^-6^
Saline (n = 4)	-35.4 (± 10.8)	-2.9 (± 0.5)	47.7 (± 16.4)	2.6 ± 0.8 × 10^-6^
Iodine (n = 4)	-27.0 (± 2.8)	-3.6 (± 0.4)	92.0 (± 15.4)	2.0 ± 0.4 × 10^-6^
ML:8 (n = 4)	-42.3 (± 4.3)	-3.2 (± 1.1)	69.8 (± 11.5)	1.2 ± 0.4 × 10^-6^

SCC and PD values were stable for the duration of the experiment. These parameters did not vary between groups. Compared with controls, electrical resistance values were slightly lower and Papp values were slightly higher in all the treated groups. None of these differences were significant.

### Different treatments are antiseptic

**Figure 1:**
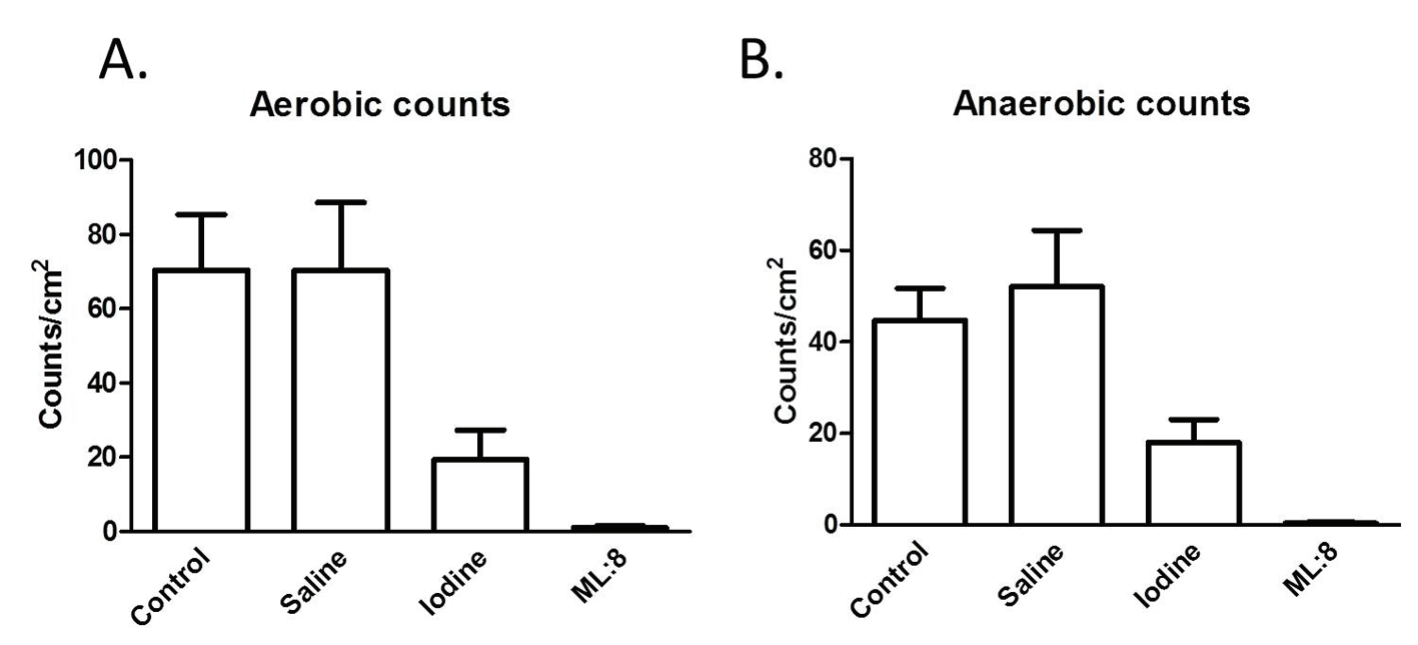
Swabs taken from the mucosal surfaces of tissues exposed to the different treatments were used to inoculate culture plates. After 48 h, numbers of colony forming units were counted as described in the “Methods” section. Results, which are similar for aerobic (A) and anaerobic (B) culture conditions indicate that ML:8 has greater efficacy than iodine at preventing bacterial growth.

Similar numbers of colony forming units were generated over 48 h of incubation in aerobic and anaerobic conditions (control; Fig. 1 A, B). The absolute numbers of colony forming units grown from specimens exposed to saline were not different from those obtained from control tissues. In contrast, iodine treatment reduced (P < 0.05) and ML:8 virtually abolished (P < 0.05) viable bacteria (Fig. 1 A, B).

### Ion transport responses

**Figure 2:**
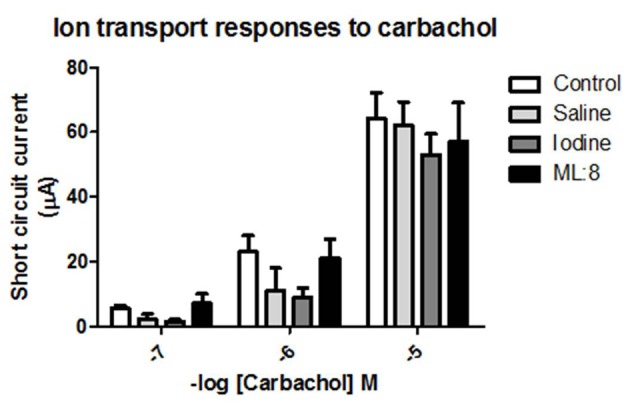
Carbachol applied to the basolateral domain of voltage-clamped human colonic mucosa evoked a rapid-onset inward short circuit current. These responses were unaffected in tissues which had been pre-exposed transiently to saline or to ML:8. Ion transport responses to carbachol in colonic sheets which had been pre-exposed to iodine were attenuated when compared to control responses by analysis of variance (P < 0.05; n = 4 - 5).

Carbachol addition to the mucosal bathing solution evoked rapid onset ion transport responses which were concentration-dependent. These responses were unaffected by pre-treatment with saline or ML:8 but were attenuated in tissues pre-exposed to povidone iodine (Fig. 2).

### Histology

Even after prolonged maintenance in Ussing chambers, tissues displayed no gross or overt histological damage. Structural integrity was largely conserved and consistent with the observed absence of gross pathology at the time of surgical resection. Crypt heights were identical in each of the control and treated groups.

## Discussion

Our results indicate that the electrophysiological profiles of tissues in the control group were in agreement with previously published values [21]. Ion transport responses to carbachol, which are due to chloride secretion via activation of muscarinic M3 receptors [22], were used as a functional marker, along with permeability to the marker molecule mannitol. Once again, the control values were consistent with those previously reported for voltage clamped human colonic mucosa [23, 24].

Pre-exposure to saline resulted in very little effect on any of the measured parameters. Aerobic and anaerobic colony counts were almost identical to control values, ion transport responses to carbachol and structural appearance of saline treated tissues were not different from controls. Exposure to saline was associated with a modest increase in permeability to mannitol and reduction in electrical resistance. Thus saline wash was relatively innocuous to the tissue and had little effect on the retrieved culturable burden. Saline is used widely as a surgical irrigant [25].

In contrast, iodine and ML:8 treatments significantly reduced culturable bacteria, almost abolishing this parameter in the case of ML:8. Povidone iodine is widely used as a surgical irrigant and its efficacy has been validated in randomized control trials [26]. ML:8 is a prototype surgical irrigant which is derived from a formulation with applications as a catheter locking solution [27], in dental hygiene [28] and gynecologically in peri-partum care in cattle [29]. These studies indicate the efficacy of ML:8 against a wide range of selected microorganims, referenced against vancomycin and also against chlorhexidine. In the current study, we selected methods with which to address a global effect on all commensal culturable bacteria, not only individual species or strains.

Ion transport responses to carbachol were unaffected after ML:8 treatment but slightly, and significantly, reduced after exposure to iodine. This is consistent with a report that povidone iodine was deleterious to human intestine even when lumenal exposure was quite brief in duration [30]. Iodine and ML:8 increased permeability to mannitol and reduced electrical resistance. These effects were quite modest. Both povidone iodine and fatty acids (which are components of ML:8) have been evaluated as excipients to enhance bioavailability of poorly water-soluble drugs by enhancing absorption [31]. Tissue appearance was not different from controls following any of the treatments. This is in keeping with the lack of cytotoxicity when ML:8 was compared with chlorhexidine in an *in vitro* study [28].

The precise mechanism(s) of action of ML:8 remain to be established. Fatty acids are antibacterial and/or antiseptic [32, 33]. For example, caprylic (C8), capric (C10) and lauric acid (C12) display antimicrobial activity [34]. Some of these act by disrupting the bacterial plasma membrane [35]. Inhibition of adhesion by amphipathic interactions may also contribute to antimicrobial efficacy. One of the components of ML:8, lecithin which was originally introduced to the formulation as an emulsifying agent, may contribute to the inhibitory effect on bacterial adhesion through surfactant action [36].

### Conclusions

We have demonstrated that brief exposure to ML:8 reduces culturable bacterial burden from human intestinal tissues harvested at the time of surgical resection. The tissues retained structural and functional integrity. This preparation of human tissue complements the established option of using tissues from germ-free animals for studies of single bacteria or known consortia of bacteria on host physiology [37-39]. Thus, under well-defined conditions, microorganisms (pathogens, commensals and/or probiotics) may be used to challenge such germ-free tissues *ex vivo*. ML:8 may have a role in reducing microbiotic burden in tissues harvested not only for ion transport or electrophysiological studies, but also for longer term culture of biopsy material for genomic, i.e. longer duration, experiments. Thus interactions between commensals and pathogens, which are critical to normal function [40], may be studied.

In addition to providing models with which to understand fundamental mechanisms of host-commensal interactions, other significant applications include using ML:8 to prevent surgical site infection or as a surgical irrigation fluid during regular abdominal procedures including natural orifice translumenal endoscopic surgery (NOTES) for which iodine or cyclohexidine, along with intravenous antibiotics has been shown to be clinically effective [41, 42]. Other applications include intervention at or around the time of catastrophic breach of mucosal integrity following trauma which may result in contamination by commensal microorganisms with high morbidity and mortality [43, 44]. Similarly, surface decontamination of the mucosal surfaces may offer palliative benefits in conditions such as pouchitis [45].
